# Single-cell RNA profiling links ncRNAs to spatiotemporal gene expression during *C. elegans* embryogenesis

**DOI:** 10.1038/s41598-020-75801-3

**Published:** 2020-11-02

**Authors:** Yan Sun, Qichao Yu, Lei Li, Zhanlong Mei, Biaofeng Zhou, Shang Liu, Taotao Pan, Liang Wu, Ying Lei, Longqi Liu, Radoje Drmanac, Kun Ma, Shiping Liu

**Affiliations:** 1 BGI Education Center, University of Chinese Academy of Sciences, Shenzhen, 518083 China; 2 BGI-Shenzhen, Shenzhen, 518083 China; 3 Shenzhen Key Laboratory of Single-Cell Omics, BGI-Shenzhen, Shenzhen, 518100 China

**Keywords:** Embryogenesis, Computational biology and bioinformatics

## Abstract

Recent studies show that non-coding RNAs (ncRNAs) can regulate the expression of protein-coding genes and play important roles in mammalian development. Previous studies have revealed that during *C. elegans* (*Caenorhabditis elegans*) embryo development, numerous genes in each cell are spatiotemporally regulated, causing the cell to differentiate into distinct cell types and tissues. We ask whether ncRNAs participate in the spatiotemporal regulation of genes in different types of cells and tissues during the embryogenesis of *C. elegans*. Here, by using marker-free full-length high-depth single-cell RNA sequencing (scRNA-seq) technique, we sequence the whole transcriptomes from 1031 embryonic cells of *C. elegans* and detect 20,431 protein-coding genes, including 22 cell-type-specific protein-coding markers, and 9843 ncRNAs including 11 cell-type-specific ncRNA markers. We induce a ncRNAs-based clustering strategy as a complementary strategy to the protein-coding gene-based clustering strategy for single-cell classification. We identify 94 ncRNAs that have never been reported to regulate gene expressions, are co-expressed with 1208 protein-coding genes in cell type specific and/or embryo time specific manners. Our findings suggest that these ncRNAs could potentially influence the spatiotemporal expression of the corresponding genes during the embryogenesis of *C. elegans*.

## Introduction

It is known that although about 90% of the eukaryotic genome is transcribed only 1–2% of the transcripts, known as messenger RNAs (mRNAs) encode proteins, while the majority of transcripts called non-coding RNAs (ncRNAs) do not encode proteins. Although the specific functions of the majority of ncRNAs remain unclear, some ncRNAs are known to play roles in translation, RNA splicing^1^, DNA replication^2^, gene regulation^3^, genome defense^4^, and chromosome structure^5^.

*Caenorhabditis elegans* is an excellent animal model to study molecular mechanisms in developmental biology because of its established cell lineage^6,7^, well-defined anatomy and genomic characteristics^8^, and completed single cell atlases^9,10^. The genome of *C. elegans* (WBcel235) harbors 20,447 protein-coding genes and 26,301 annotated ncRNAs, of which only approximately 1300 are thus far known to play roles in various biological processes^11^, including structural components such as tRNAs, rRNAs, small nucleolar RNAs (snoRNAs) and small nuclear RNAs (snRNAs), and regulatory components such as microRNAs (miRNA)^12,13^ and long ncRNAs (lncRNAs)^14,15^. The majority of ncRNAs are thought to be unfunctional^8,16,17^.

Recent studies have shown that some ncRNAs are important for embryogenesis in human and mouse^18^, such as lncRNAs (*TUNA* and *HOTAIR*), and miRNA *miR-145*^19,20^. It is known that during *C. elegans* embryo development, numerous genes in each cell are uniquely and spatiotemporally expressed, causing the cell to differentiate into distinct cell types and tissues^21,22^, and that ncRNAs such as *lin-4*, *let-7* and *lep-5* can influence post-embryonic development^12^, larva transitions^13,23^, and sexual maturations^14^, respectively. However, little is known whether ncRNAs may influence the unique and spatiotemporal gene expression during the embryogenesis of *C. elegans*. To address these questions, we analyzed 1031 whole transcriptomes of cells from mixed stages obtained from multiple *C. elegans* embryos, using marker-free full-length high-depth single-cell RNA sequencing (scRNA-seq) technique. We detected a total of 20,436 protein-coding genes and 9843 ncRNAs in these cells, and identified 94 ncRNAs that potentially could impact the spatiotemporal expression of specific genes during the embryogenesis of *C. elegans*.

## Results

### Quantity of expressed protein-coding genes and ncRNAs vary vastly in embryonic cells

We obtained the full-length transcriptomes of 1031 high quality embryonic cells from multiple embryos of mixed-stages using a conventional marker-free scRNA-seq technique^24^. We obtained 1.62 billion qualified sequencing reads (median 1.47 M per cell, range 0.3–62.8 M per cell, see “Methods”) and totally detected 20,436 out of 20,447 (99.95%) protein-coding genes and 9843 (transcript length range 43–56,600 bp) out of 10,679 (92.17%, excluding miRNA and piRNA) ncRNAs from 1031 embryonic cells, including 99 antisense RNAs, 169 long intervening non-coding RNAs (lincRNAs), 22 rRNAs, 338 snoRNAs, 1546 pseudogenes, 126 snRNAs, 571 tRNAs and 6972 ncRNAs of unknown types (TPM > 1, Table 1). We did not detect any miRNAs (transcript length range 18–50 bp) and piRNAs (21 bp) for they do not have poly-a tails and scRNA-seq depends on poly-A tails.

**Table 1:** Summary of detected genes.

Gene types	Genes detected per cell Median (min–max)	Total genes detected	Genes annotated in Ensembl release 80	Detect ratio (%)
Protein coding	8746 (1216–13,862)	20,431	20,447	99.92
**ncRNA**				
Antisense	18 (0–45)	99	99	100.00
lincRNA	33 (4–94)	169	169	100.00
rRNA	6 (6–21)	22	22	100.00
snoRNA	28 (4–72)	338	345	97.97
pseudogene	132 (24–644)	1546	1590	97.23
snRNA	6 (0–59)	126	130	96.92
tRNA	5 (0–112)	571	637	89.64
Unknow^a^	223 (37–1022)	6972	7687	90.70

^a^Unknow: ncRNAs of unknown types (median length: 140 bp, range 17–2525 bp).

**Figure 1:**
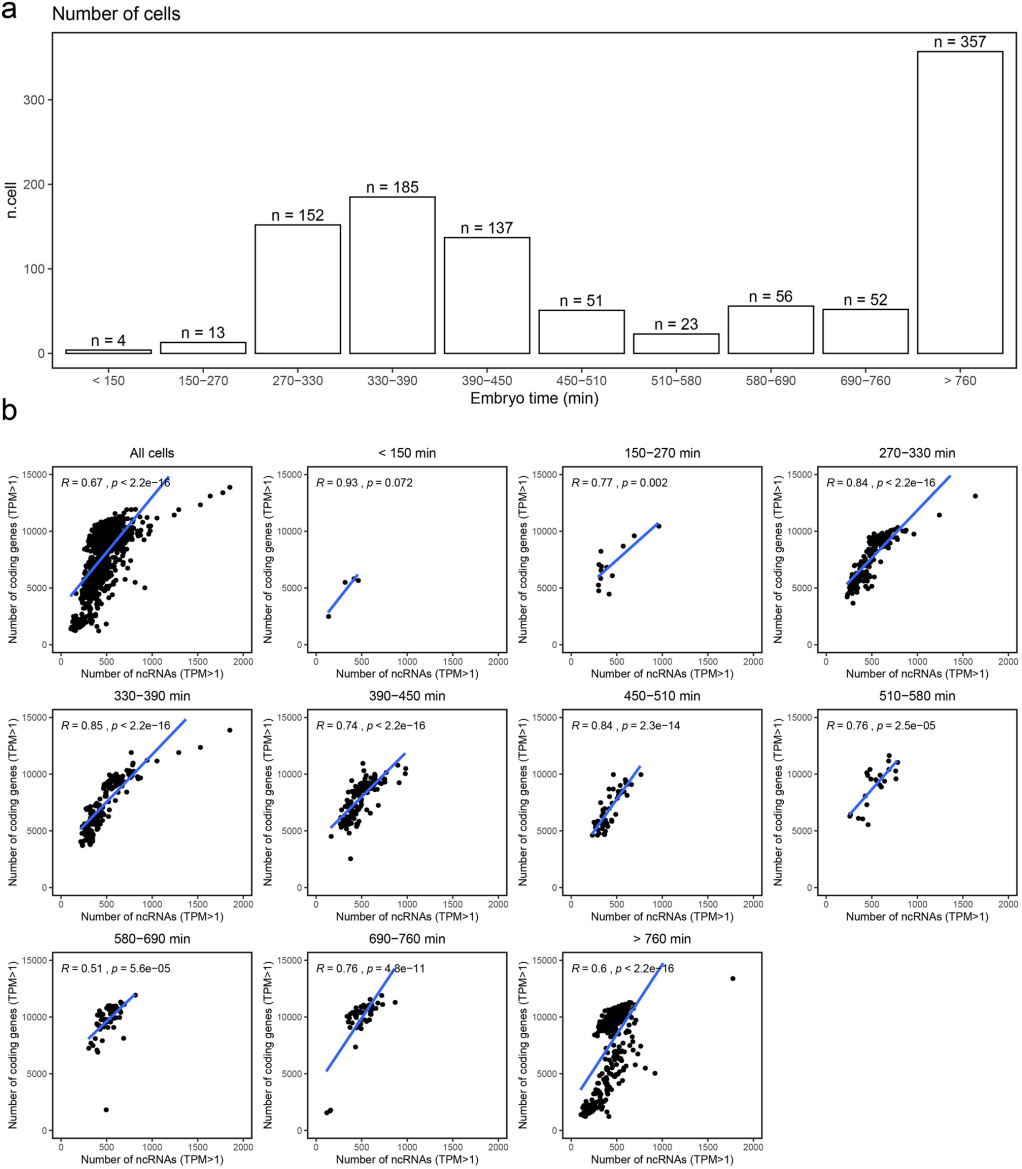
An outline of the 1031 embryonic cells and the detected genes pre cell by time intervals. (**a**) Number of cells within each time interval. (**b**) Pearson correlations between detected protein-coding genes and ncRNAs per cell in each time interval.

Though embryos can be synchronized, their developmental stages may still be dispersed to a certain extent^10,25^. To determine the embryonic stages of the cells, we estimated the ‘embryo time’ of each cell by calculating the Pearson correlation between single cell transcriptome profiling and those of whole embryos collected at multiple time points^26^, a standard method used by Packer et al.^10^. The cells were subsequently divided into 10 embryo-time intervals between < 150 min and > 760 min, according to the expression patterns of time-dependent genes^10,26^. Notably, the majority of cells came from embryo time intervals between 270 and 450 min (n = 474), and over 760 min (n = 357, Fig. 1a). We noticed that the quantity of expressed protein-coding genes and ncRNAs in each cell varied immensely within an embryo-time interval and between different embryo-time intervals (Table 2), which was also observed by Packer et al.^10^. However, we detected many more protein-coding genes per cell as compared to those of other single-cell studies of *C. elegans* reported by Packer et al., and others done with Drop-based scRNA-seq platforms^9,10^ (Table 2).

**Table 2:** Comparison of protein-coding genes and ncRNAs detected per cell in different embryo time intervals.

Time intervals	Protein-coding genes per cell Mean (min–max)		ncRNAs per cell Mean (min–max)
	We detected	Packer et al.^10^ detected	
< 150	4863.8 (2478–5812)	1958.3 (333–5087)	327.5 (131–457)
150–270	6975.9 (4449–10,437)	1054.6 (287–4182)	432.2 (293–956)
270–330	7658.6 (3648–13,113)	937 (250–4983)	500.7 (223–1631)
330–390	7342.7 (3697–13,862)	847.5 (239–4410)	475.9 (211–1846)
390–450	7849.5 (2543–10,943)	758.3 (219–4271)	480.5 (159–977)
450–510	6843.5 (4609–9953)	733 (213–4494)	416 (227–759)
510–580	8949.6 (5528–11,622)	673.6 (178–3883)	532.3 (253–778)
580–690	9724.1 (1816–11,934)	639.2 (136–3,819)	513.9 (298–810)
690–760	9781 (1549–11,893)	912.1 (136–3,695)	490 (111–861)
> 760	7654.7 (1216–13,384)	1409.2 (307–4160)	433.2 (102–1769)

To investigate the expression profiling of protein-coding genes and ncRNAs during embryogenesis, we analyzed the quantity of protein-coding genes and ncRNAs expressed in cells of each embryo-time interval. We found moderate (R = 0.51, P = 5.6e−05) to high (R = 0.85, P < 2.2e−16) Pearson correlations between the number of protein-coding genes and that of ncRNAs in cells through all embryo-time intervals (Fig. 1b).

### Determination of cell types

**Figure 2:**
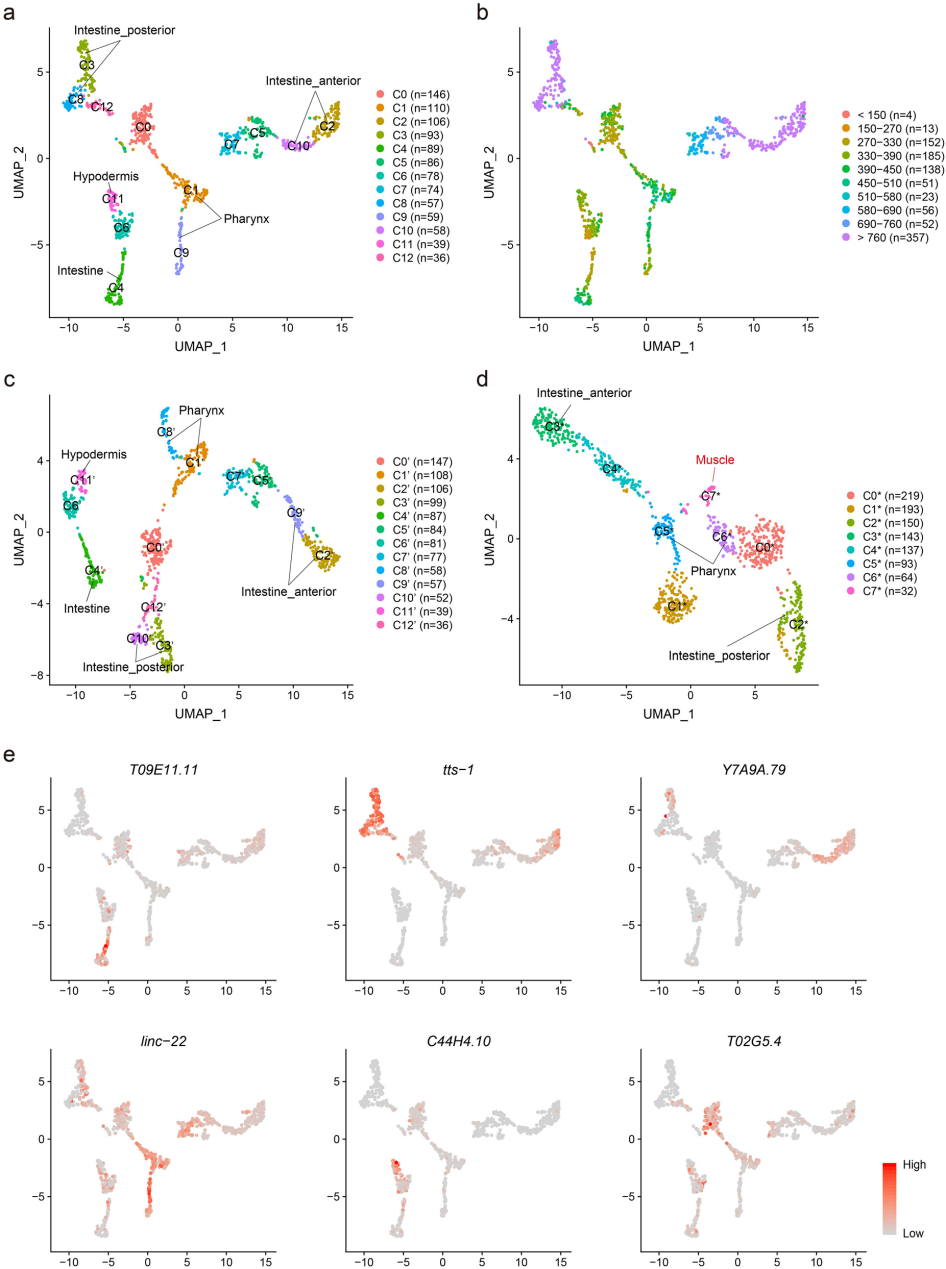
Clustering of the embryonic cells. (**a**, **c**, **d**) Clustering 1031 embryonic cells using combined protein-coding genes and ncRNAs (**a**), using protein-coding genes alone (**c**), and using ncRNAs alone (**d**). (**b**) Clustering cells using combined protein-coding genes and ncRNAs, and labelling cells with embryo times. (**e**) Feature plots of newly identified ncRNA markers: *T09E11.11* (early and middle embryonic intestinal cells), *tts-1* (late embryonic posterior intestinal cells), *Y7A9A.79* (late embryonic anterior intestinal cells), *linc-22* (pharyngeal cells), *C44H4.10* (hypodermal cells), *T02G5.4* (early embryonic cells).

To analyze the identities and functions of the 1031 embryonic cells, we first clustered and visualized the cells based on the expression profiling of protein-coding genes and ncRNAs detected in each cell, using the FindClusters function and the Uniform Manifold Approximation and Projection (UMAP) algorithm from the Seurat R package^27^. As a result, the 1031 cells were divided into 13 clusters (C0–C12, Fig. 2a), including intestinal cells (marker genes: *irg-7*, *ZC204.12*, *spp-5*, *ifb-2*, *pept-1*, *F56C9.7*, *aqp-4*, *cyp-35A2*, *pyk-2*) in C4,C3,C8,C2 and C10, pharyngeal cells (marker genes: *ceh-2*, *hlh-6*, *phat-2*, *tni-4*, *spp-7*, *sulp-3*, *abu-14*) in C1 and C9, hypodermal cells (marker genes: *elt-3*, *vab-3*, *mlt-11*, *acn-1*, *ifa-3*, *lin-26*, *lpr-5*) in C11, early (embryo time < 390 min) embryonic cells in C0 and C6 , and cells of unknown types in C5, C7 and C12 (Fig. 2a). Intriguingly, the intestinal cells were divided into five clusters, i.e. early (< 390 min) and middle (390–690 min) embryonic intestinal cells (C4), late (> 690 min) embryonic posterior intestinal cells (C3 and C8, marker genes: *irg-7*, *pbo-4*) and late embryonic anterior intestinal cells (C2 and C10, marker gene: *ceh-37*) (Fig. 2a,b).

To search for protein-coding genes and ncRNAs that were cell-type-specifically and/or temporally expressed during the embryogenesis of *C. elegans*, we used the FindAllMarkers function to obtain the top 10 highly expressed (top tenfold change, p.adjust < 0.05) and cluster-specific protein-coding genes and ncRNAs that were expressed in at least 75% cells of the cluster. We subsequently identified 33 new markers, including 22 protein-coding genes and 11 ncRNAs that were highly expressed in embryo time and/or cell type-specific manners (Fig. 2e, Supplementary Figs. S1, S2). For example, six protein-coding genes (*acdh-1*, *Y38F1A.6*, *inx-15*, *nep-17*, *cpz-1* and *nep-22*) and one ncRNA (*T09E11.11*) were specifically expressed in early and middle embryonic intestinal cells (Fig. 2e, Supplementary Figs. S1, S2). Besides, two protein-coding genes (*T21H3.1* and *asp-5*) and two ncRNAs (*tts-1* and *B0250.3*) were distinctively expressed in late embryonic posterior intestinal cells, whereas nine protein-coding genes (*nlp-28*, *nspe-1*, *fipr-2*, *F57F5.1*, *osm-11*, *grl-21*, *T04F8.8*, *cnc-11* and *F45E4.5*) and one ncRNA (*Y7A9A.79*) were exclusively expressed in late embryonic anterior intestinal cells (Fig. 2e, Supplementary Figs. S1, S2). In addition, one protein-coding gene (*F56C9.8*) and one ncRNA (*linc-22*) were specifically expressed in pharyngeal cells, while two ncRNAs (*C44H4.10* and *K02E7.5*) were specifically expressed in hypodermal cells (Fig. 2e, Supplementary Figs. S1, S2). Interestingly, we identified four protein-coding genes (*clec-87*, *his-24*, *lbp-1* and *mig-6*) and four ncRNAs (*T02G5.4*, *C33D9.5*, *F07H5.5* and *anr-1*) exclusively expressed in 354 early (< 390 min) embryonic cells (Fig. 2e, Supplementary Figs. S1, S2), suggesting that the expression of these protein-coding genes and ncRNAs can be used as markers for identifying early (< 390 min) embryonic cells.

Given that some ncRNAs are cell type-specifically expressed, we further investigated whether other ncRNAs also expressed in cell type-specific manners. We re-clustered the cells with the same clustering parameters, but using either protein-coding genes (Fig. 2c) or ncRNAs (Fig. 2d), respectively. We found that the 1031 cells were also clustered into 13 clusters (C0′–C12′, Fig. 2c) when using protein-coding genes alone. However, when using ncRNAs alone, the 1031 cells were clustered into eight clusters (C0*–C7*, Fig. 2d), perhaps partially due to smaller quantity of ncRNAs detected per cell compared to that of protein-coding genes (Table 2). We noticed that ncRNA-based clustering formed a new cluster (C7*, median of expressed ncRNAs per cell = 400, median ratio of mitochondrial reads = 1.2%) that comprised 32 high-quality cells, which were dispersed in five clusters (C5, C7, C6, C1 and C3) when using whole transcriptome-based clustering strategy (Supplementary Fig. S3a). Further analysis indicated that protein-coding genes (*myo-3, act-3*, *act-2*, *act-1*, *mup-2*, *unc-27*, *tni-1*, *mlc-2*, *unc-15* and *lev-11*) involved in cytoskeleton organization, muscle development, muscle system process, sarcomere organization, were highly expressed in the 32 cells of the new cluster (C7*), suggesting that they were muscle cells. Interestingly, we found that the prime elements that separated these muscle cells from others were coexistence of distinctively (top 10) high expressions of ncRNAs *T04C12.26*, *F07H5.3*, *T04C12.17*, *pgp-15* and substantially low expressions of ncRNAs *M163.16*, *C14B9.11*, *T02G5.4*, *W06H8.5*, *tts-1* (Supplementary Fig. S3b, C7*), indicating that these ncRNAs are expressed in cell type-specific manners, and important for embryonic muscle development of *C. elegans*. These findings also suggest that ncRNA-based clustering can be used for identifying cell types as a complementary strategy to the protein-coding gene-based clustering strategy.

### Expressions of ncRNAs and their corresponding protein-coding genes are highly corelated in spatiotemporal manners during embryogenesis

Co-expressions of ncRNAs and protein-coding genes are frequently used to identify functional ncRNA/protein-coding gene relationships (Guilt by Association)^28–30^. To investigate whether the expression of ncRNAs can influence protein-coding gene expressions, we searched for ncRNAs that were co-expressed with protein-coding genes (see “Methods”). We identified 94 ncRNAs (of which 88 were thought to be unfunctional according to WormBase^31^) that were co-expressed (R ranged 0.6 to 0.95 and − 0.6 to − 0.75) with a total of 1208 protein-coding genes (including *let-502*, *set-1*, *lat-1*, *sdn-1*, *xnp-1*, *nmy-1*, *cdl-1*, *let-418* and *alg-2*, known to be important in the embryogenesis of *C. elegans*^32–40^, Supplementary Table S1, Supplementary Fig. S4) in cells of different types (Supplementary Fig. S5).

We identified a number of ncRNAs that were individually co-expressed with multiple protein-coding genes. Notably, 23 ncRNAs were individually co-expressed with more than 50 protein-coding genes (Supplementary Table S1), and 9 ncRNAs (*Y7A9A.79*, *M163.16*, *T05E11.9*, *anr-24*, *F41E7.20*, *linc-111*, *tts-2*, *C33D9.5*, *rrn-3.1*, Supplementary Table S1) were individually co-expressed with more than 100 protein-coding genes. Especially, ncRNAs *M163.16* (Supplementary Fig. S6) and *Y7A9A.79* (Supplementary Fig. S7) were individually co-expressed with more than 200 protein-coding genes (Supplementary Table S1). In addition, we identified 71 ncRNAs, of which some seemed to act conjointly, co-expressed with specific sets of protein-coding genes (Supplementary Fig. S4, Supplementary Table S2). For instance, ncRNAs *T04C12.26* and *T04C12.17* (Supplementary Fig. S3b, Supplementary Table S2) were co-expressed in muscle cells (Supplementary Fig. S3b) with a set of protein-coding genes *act-3*, *act-1*, *act-2*, *act-4* and *mlc-3*, known to be involved in muscle process^41,42^. We found eight ncRNAs that were co-expressed with the same protein-coding genes in opposite manners. For example, protein-coding genes *clec-52*, *ins-11*, known to be involved in immune response according to WormBase^31^, were positively co-expressed with ncRNA *Y7A9A.79* (Fig. 2e) but negatively co-expressed with rRNAs *rrn-1.1*, *rrn-1.2*, *rrn-2.1*, *rrn-3.1* (Supplementary Fig. S8, Supplementary Table S2). Likewise, protein-coding genes *tnc-2*, *phat-4*, *phat-5*, known to be involved in pharyngeal muscles and glands^31^, were positively co-expressed with ncRNA *T04C12.30* but negatively co-expressed with rRNA *F54D7.7* (Supplementary Table S2).

**Figure 3:**
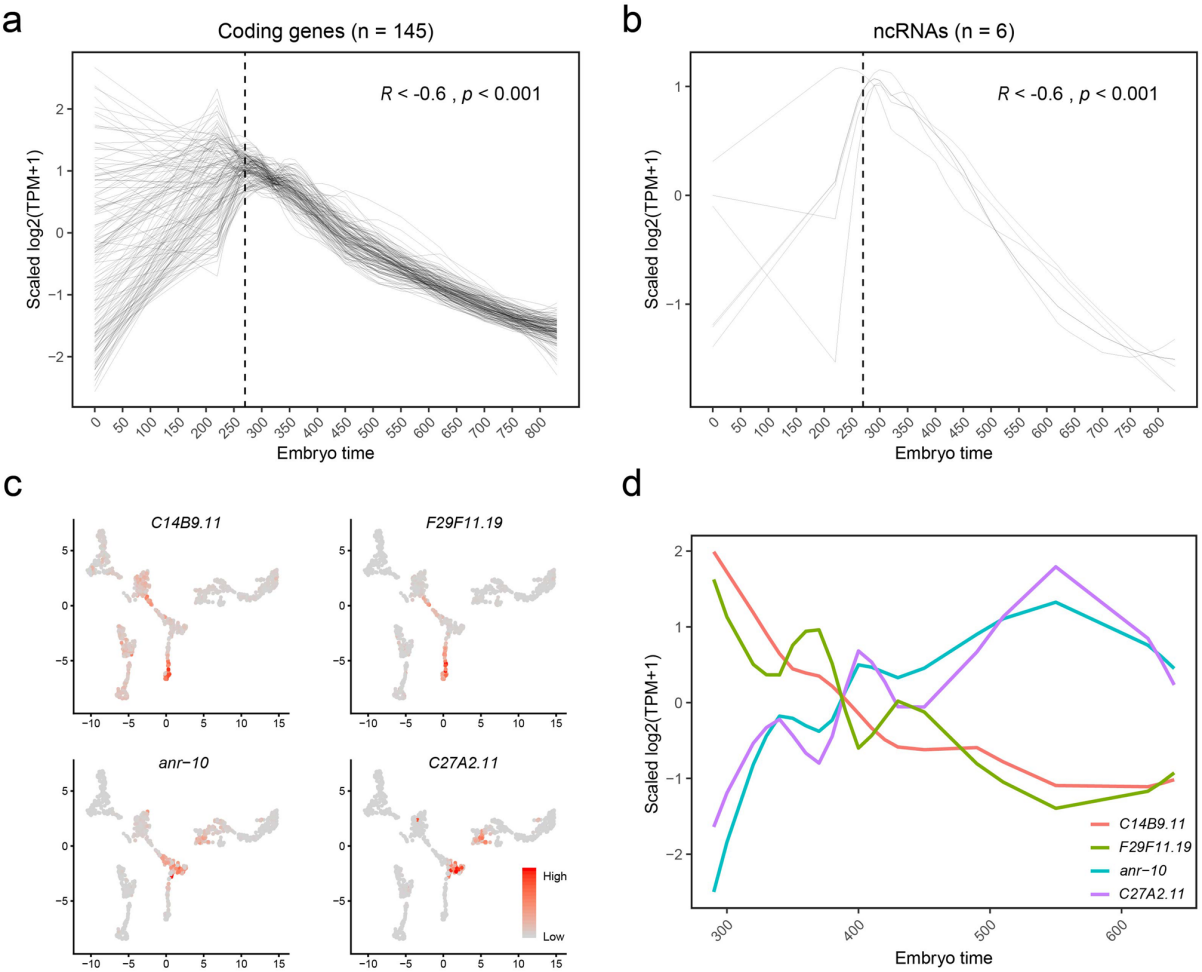
ncRNAs and protein-coding genes involving embryo and organ development. (**a**, **b**) Smoothed expressions (scaled log2-TPM, loess regression, span = 0.5) of 145 protein-coding genes (**a**) and 6 ncRNAs (**b**) along embryo times. The dashed line labels 270 min before which there are only 17 cells, and after which there are 1014 cells. (**c**) Feature plots showing expression levels of the pharyngeal expressed ncRNAs *C14B9.11*, *F29F11.19*, *anr-10* and *C27A2.11*. (**d**) Smoothed expressions (scaled log2-TPM, loess regression, span = 0.5) of the pharyngeal expressed ncRNAs along embryo times. ncRNAs *C14B9.11* and *F29F11.19* are expressed earlier, and ncRNAs *anr-10* and *C27A2.11* later in pharynx.

During the embryogenesis of *C. elegans*, the majority of cell divisions and differentiations occur at relatively earlier stages (about < 390 min)^6^. We discovered that the expression levels of 145 protein-coding genes (excluding known maternally deposited transcripts^43,44^) and six ncRNAs (*anr-1*, *rrn-1.1*, *rrn-1.2*, *rrn-2.1*, *rrn-3.1* and *C14B9.11*) continuously decreased as embryos developed from 270 to 830 min (Fig. 3a,b), raising a question of whether the changes in the expression of these ncRNAs would impact the expression of the 145 protein-coding genes at early embryo stages. GO enrichment analysis revealed that these 145 protein-coding genes were involved in multiple biological processes including chromosome organization, mitotic cell cycle, embryo development, mRNA processing, and body morphogenesis, respectively.

We noticed that some ncRNAs were co-expressed with protein-coding genes in the same organ but at different embryonic stages. For example, ncRNA *C14B9.11* and protein-coding genes *cam-1*, *cdh-4*, *ina-1*, *mab-20*, *ebax-1*, *sdn-1*, *unc-37*, *vab-1*, *ham-1*, *hmr-1*, and ncRNA *F29F11.19* and protein-coding gene *ceh-22* were co-expressed in early embryonic (< 390 min) pharyngeal cells, respectively (Fig. 3c,d). Protein-coding genes *cam-1*, *cdh-4*, *ina-1*, *mab-20*, *ebax-1*, *sdn-1*, *unc-37*, *vab-1*, *ham-1* and *hmr-1* are known to be involved in neurogenesis and axon guidance^31^, while *ceh-22* gene is required for normal pharynx development^45^. In contrast, ncRNAs *anr-10* and *C27A2.11*, and five abu/pqn paralog group (APPG) genes *abu-14*, *abu-11*, *pqn-29*, *pqn-63*, *pqn-74* were co-expressed in middle embryonic (390–690 min) pharyngeal cells (Fig. 3c,d). It has been reported that *abu-14*, *abu-11*, *pqn-29*, *pqn-63*, *pqn-74* are important for the formation and function of pharyngeal cuticle during embryogenesis^22^. The findings of the spatiotemporally correlated expressions of the aforementioned ncRNAs and their corresponding protein-coding genes suggest a possibility of regulatory functions of these ncRNAs during the organogenesis of *C. elegans*.

## Discussion

In this study, we have detected 9843 ncRNAs from 1031 *C. elegans* embryonic cells, and identified a total of 33 new markers for specific cell types (embryonic stages), including 22 protein-coding genes and 11 ncRNAs. We have shown that the quantity of expressed protein-coding genes and ncRNAs varied dramatically in different types of cells at the same embryonic stage (or within an embryo-time interval), and in the same type of cells at different embryonic stages (between different embryo-time intervals).

We have demonstrated that at least 94 ncRNAs (88 of which were thought to be unfunctional) were co-expressed with 1208 protein-coding genes (R ranged 0.6–0.95 and − 0.6 to − 0.75) in cell type specific and/or embryo time specific manners. We have demonstrated that 145 protein-coding genes (excluding known common maternally deposited coding genes in *C. elegans*^43,44^) and 6 ncRNAs whose expression levels gradually decreased as embryos developed, raising a question of whether changes in the expression of these ncRNAs would impact the changes in the expression of the corresponding protein-coding genes during embryogenesis. Furthermore, we have shown that seven pairs of ncRNAs/protein-coding genes were highly co-expressed (R > 0.9, p = 0, Supplementary Table S1) in different cell types and tissues, including *anr-1*/*set-1* (in early embryonic cells), *T04C12.26*/*act-3* (in muscle cells), *anr-10*/*phat-2* and *C27A2.11*/*C27A2.5* (in middle embryonic pharyngeal cells), *B0250.3*/*rpl-2* (in late embryonic posterior intestinal cells), *D1054.23*/*D1054.18* (in late embryonic anterior intestinal cells), and *C08B6.17*/*C08B6.4* (in C7 cells of unknown types), raising a possibility of regulatory impacts of these ncRNAs on the corresponding protein-coding genes during the embryogenesis of *C. elegans*.

Deep-sequencing studies have revealed that rRNAs can be split into small rRNA-derived RNAs (srRNAs)^46^, some of which regulate gene expressions acting as miRNAs^47^. In zebrafish, Locati et al. have identified srRNAs, and found miRNA-like srRNAs might function in embryogenesis by GO enrichment analysis of putative target genes^48^. In *C. elegans*, we are the first to show potential regulatory functions of rRNAs (*rrn-1.1*, *rrn-1.2*, *rrn-2.1*, *rrn-3.1*, Supplementary Table S1) in embryogenesis. However, it remains to be answered whether these rRNAs can be split into srRNAs in *C. elegans*.

We have induced a ncRNA-based clustering strategy as a complementary strategy to the protein-coding gene-based clustering strategy for single-cell classification. We have demonstrated that the ncRNA-based clustering strategy successfully pulled scattered muscle cells together into one cluster.

Our findings of the spatiotemporally expressions of the 94 ncRNAs and their correlated protein-coding genes suggest potential regulatory roles of these ncRNAs during the embryogenesis of *C. elegans*. It is worth noting that since we were not able to detect miRNAs and piRNAs for technical reasons, we cannot rule out the possibility of whether miRNAs and piRNAs were also involved in some of the changes in protein-coding gene expression that we observed. However, our findings warrant further studies of detailed roles of the 94 ncRNAs in the embryogenesis of *C. elegans*.

## Methods

### Sample preparation, library construction and sequencing

The wild-type nematode N2 strain was collected from Caenorhabditis Genetics Center (CGC, Minneapolis, MN 55455, USA). Synchronous populations of embryos of *C. elegans* were obtained according to the protocols of *WormBook*^25^. Briefly, *C. elegans* were grown on NEP plates seeded with NA22 bacterial at 20–25 ℃ for several generations. Gravid adults were dissolved in an alkaline solution to obtain sterile eggs. The mixed-stage embryonic cells were prepared by digesting the eggs with chitinase, and via pipetting to dissociate the egg shells^49,50^. Afterwards, the living single cells from mix-stage embryos were sorted and collected by flow cytometry. Single cell cDNA was prepared according to the protocols of MIRACLS^51^. For library construction, we applied the TN5 library building method we previously developed for Ion Proton sequencing^52^, which is similar to SMART-seq2^53^, except that we optimized the reaction temperature and time for the customized primers and adapters. Finally, single-end reads (a median length of 110 bp, ranging 20–400 bp) were generated on Ion PI Chip version 3 using Ion PI Hi-Q Sequencing 200 Kit from Ion Proton sequencer^24^.

### Transcriptome data processing

The raw sequences of Ion Proton System stored in BAM files^54^ were first converted into FASTQ format using bedtools (version 2.20.1)^55^. We then used cutadapt (version 1.8)^56^ to remove the adapter sequences and short reads (< 20 bp), and TMAP (version 3.6.40) to align the sequences to the genome (WBcel235). Finally, we used Samtools (version 1.3.1)^54^ to build the index of each BAM file.

### Quality control and gene expression profiling

Quality control was performed at both genomic and transcriptomic levels using QualiMap (version 2.2.1)^57^, in which the gene annotation was Ensembl release 80. The median clean base of each embryonic cell is 539.08 mega base pairs (Mbp). The median uniquely mapped reads are 4.03 Mbp. The mean mapping ratio is 78.94%, which implies a high sequencing quality. The mean length of clean reads is 111 bp. On average, more than 80% of clean reads are longer than 50 bp. The reads mapped to exonic, intronic and intergenic regions are 71.70%, 19.44%, and 8.86%, respectively. We used Salmon (version 0.8.2)^58^ to calculate read count and transcripts per million (TPM)^59^ to quantify gene expression at transcript level.

### Single cell clustering and cell type identifications

Cells were clustered using the Seurat R package (version 3.1.1)^27^, and the read count matrix was used. Read counts were first normalized with the scale factor equal to 10,000 and then natural-log transformed. All genes were used as variable features to run principal component analysis (PCA). The top 20 PCs were used to run UMAP and to cluster cells with the resolution equal to 1.0. The cluster-specific expressed genes were found using the FindAllMarkers function with the min percent of expressed cells set to 0.75 and the log fold change threshold set to 0.25 (fold change > e^0.25^), and the "wilcox" test was used, we chose the top 10 markers with the largest fold changes. Parameters were the same for the FindMarkers function, when calculating differential expressed genes. We identified cell types according to markers published by Packer et al.^10^ and markers annotated in WormBase^31^. We also projected cells onto the *C. elegans* embryo single-cell atlas published by Packer et al.^10^ using the FindTransferAnchors function with protein-coding genes and with the parameter dims = 1:30, k.anchor = 5, k.filter = 200, and validated the cell types we identified.

### Differential expressed gene analysis

We calculated differential expressed genes between cell groups using the FindMarkers function from the Seurat R package (version 3.1.1)^27^. Parameters were the same as the FindAllMarkers function.

### Estimating embryo times

Embryo times were estimated as Packer et al. described in their paper^10^. Pearson correlations was computed between log-scaled single cell data and bulk data which were collected at different embryo times^26^ using the time variable genes^10^. By fitting a loess regression curve with the parameter span = 0.75 and finding its maximal point, we assigned each cell with its most correlated bulk time point.

We also projected the 1031 cells onto the almost complete *C. elegans* embryo single-cell atlas^10^ using the FindTransferAnchors function with the time variable genes and with the parameter dims = 1:30, k.anchor = 5, k.filter = 200. And we obtained similar embryo times for each cell from this function.

### Correlations between genes’ expressions and selecting potential regulatory ncRNAs

We first log scaled the TPM matrix by calculating log2 (TPM + 1). And the log2 (TPM + 1) matrix was used to calculate Pearson correlations and p-values between genes’ expressions with the rcorr function from the Hmisc R package (version 4.3.0). And we kept gene pairs, of which the absolute values of correlation (R) were greater than 0.6 and of which the p value were less than 1e−5.

For each ncRNA, we calculated its Pearson correlations of expression with all the protein-coding genes. For the expression of a gene can be influenced by biological factors (i.e. regulation) or by stochastic non-biological disturbances. In principle, if a ncRNA can influence gene expression it should be able influence the expression of more than one gene. To exclude changes in a gene expression possibly due to stochastic non-biological disturbances, we kept ncRNAs only if they were either positively co-expressed with at least 4 protein-coding genes (R > 0.6, p = 0) or negatively co-expressed with at least 4 protein-coding genes (R < − 0.6, p = 0). As a result, we obtained the 94 ncRNAs.

### GO enrichment analysis of gene sets

GO enrichment analysis was performed using the enrichGO function from the clusterProfiler R package (3.10.1)^60^, with the org.Ce.eg.db database (version 3.7.0).

### Statistical test and plotting

Two-tailed t test was used to examine read counts, and expressed genes. All the figures were generated in R Software^61^.

## Supplementary information

Supplementary Information 1.

Supplementary Information 2.

Supplementary Information 3.

Supplementary Information 4.

## Data Availability

All the raw sequences were deposited in the National Center for Biotechnology Information (NCBI) and can be accessed in the Short Read Archive (SRA, accession: SRP112706) linking to BioProject accession number PRJNA393602. The data have also been deposited into CNGB Sequence Archive (CNSA: https://db.cngb.org/cnsa/) of CNGBdb with accession number CNPhis0002992.
