# Supplementary material for: Single-cell RNA profiling links ncRNAs to spatiotemporal gene expression during C. elegans embryogenesis
Source: Sci Rep. 2020 Nov 2;10:18863. doi: 10.1038/s41598-020-75801-3 (PMC7606524; doi:10.1038/s41598-020-75801-3)
Supplement: Supplementary file 1 — Supplementary Information 1. [file 41598_2020_75801_MOESM1_ESM.pdf]

Single-cell RNA profiling links ncRNAs to spatiotemporal gene expression during *C. elegans* embryogenesis

Yan Sun [1, 2, 3, †], Qichao Yu [2, 3, †], Lei Li [1, 2], Zhanlong Mei [2], Biaofeng Zhou [1, 2, 3], Shang Liu [1, 2, 3], Taotao Pan [1, 2, 3], Liang Wu [1, 2, 3], Ying Lei [2, 3], Longqi Liu [2, 3], Radoje Drmanac [2 *],Kun Ma [2, 3 *], Shiping Liu [1, 2, 3 *]

[†] Equal contribution

* Correspondence: rdrmanac@completegenomics.com (Radoje Drmanac), makun1@genomics.cn (Kun Ma), liushiping@genomics.cn (Shiping Liu)

Affiliations

[1] BGI Education Center, University of Chinese Academy of Sciences, Shenzhen 518083, China

[2] BGI-Shenzhen, Shenzhen 518083, China

[3] Shenzhen Key Laboratory of Single-Cell Omics, BGI-Shenzhen, Shenzhen 518100, China

Emails:

Shiping Liu: liushiping@genomics.cn; Kun Ma: makun1@genomics.cn; Radoje Drmanac: rdrmanac@completegenomics.com; Yan Sun: sunyan4@genomics.cn; Qichao Yu: yuqichao@genomics.cn; Lei Li: lilei2@genomics.cn; Zhanlong Mei: meizhanlong@genomics.cn; Biaofeng Zhou: zhoubiaofeng@genomics.cn; Shang Liu: liushang@genomics.cn; Taotao Pan: pantaotao@genomics.cn; Liang Wu: wuliang@genomics.cn; Ying Lei: leiying1@genomics.cn; Longqi Liu: liulongqi@genomics.cn

Supplementary figures

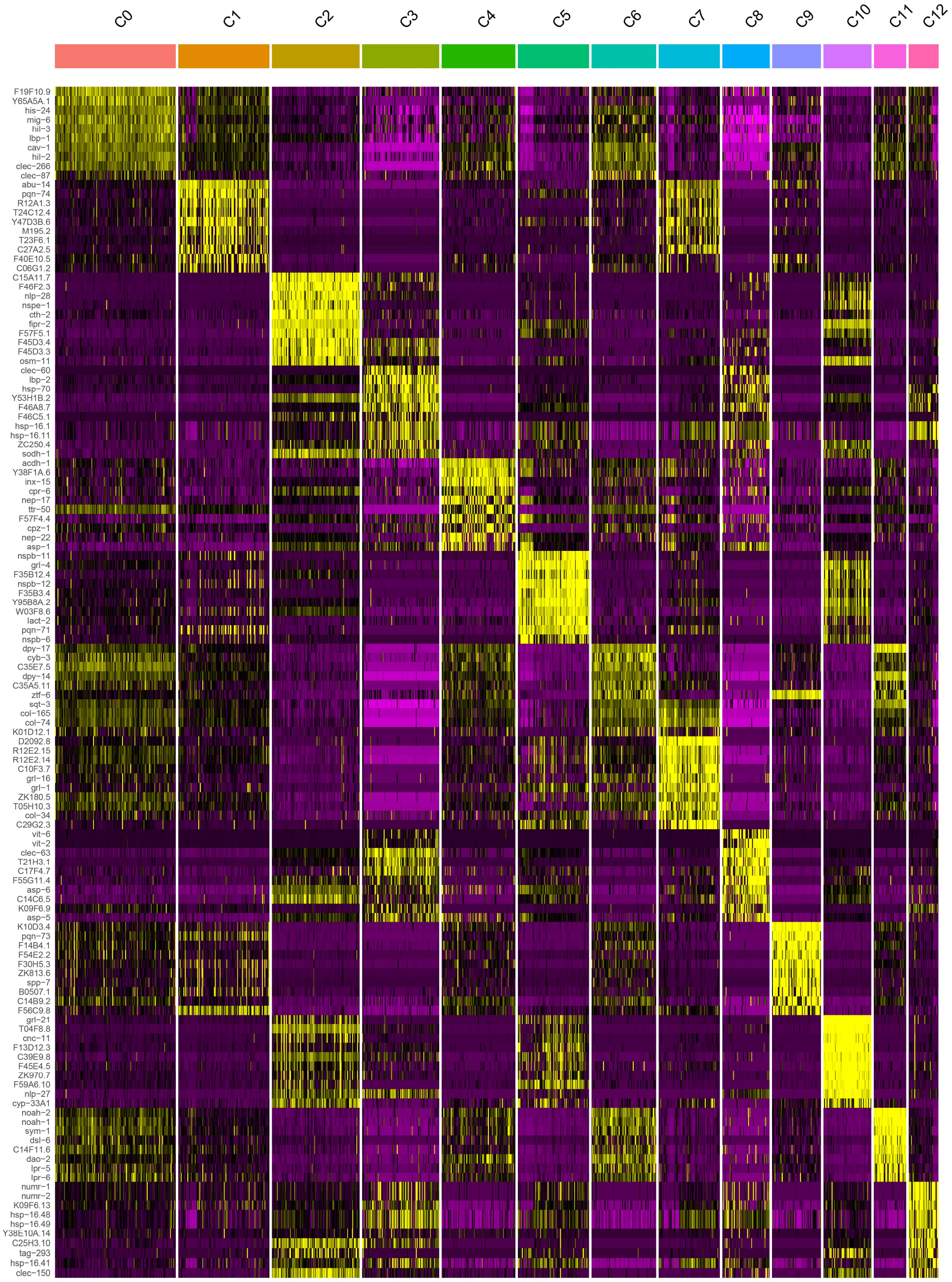

Figure S1. Heatmap of the top 10 protein-coding markers.

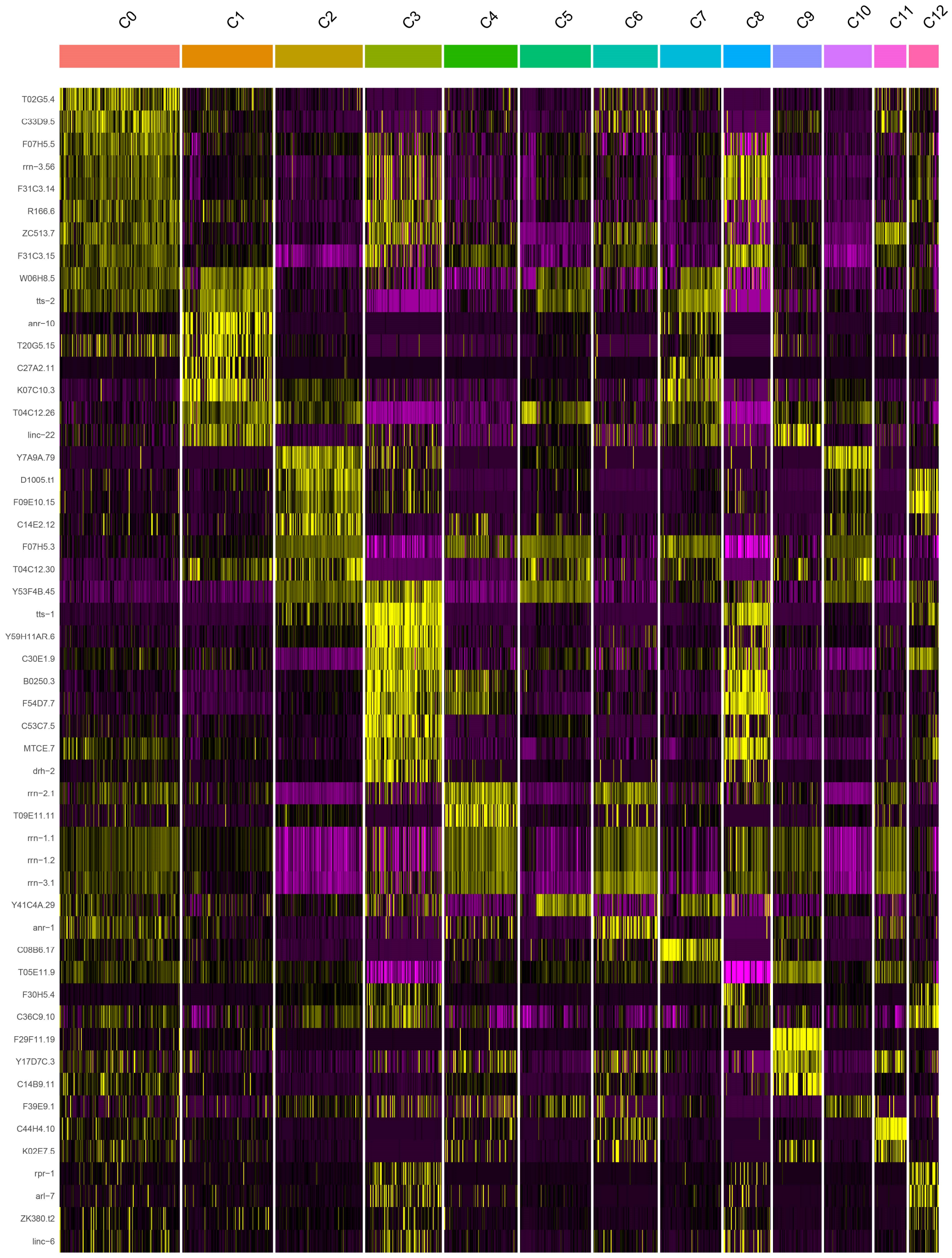

Figure S2. Heatmap of the top 10 ncRNA markers.

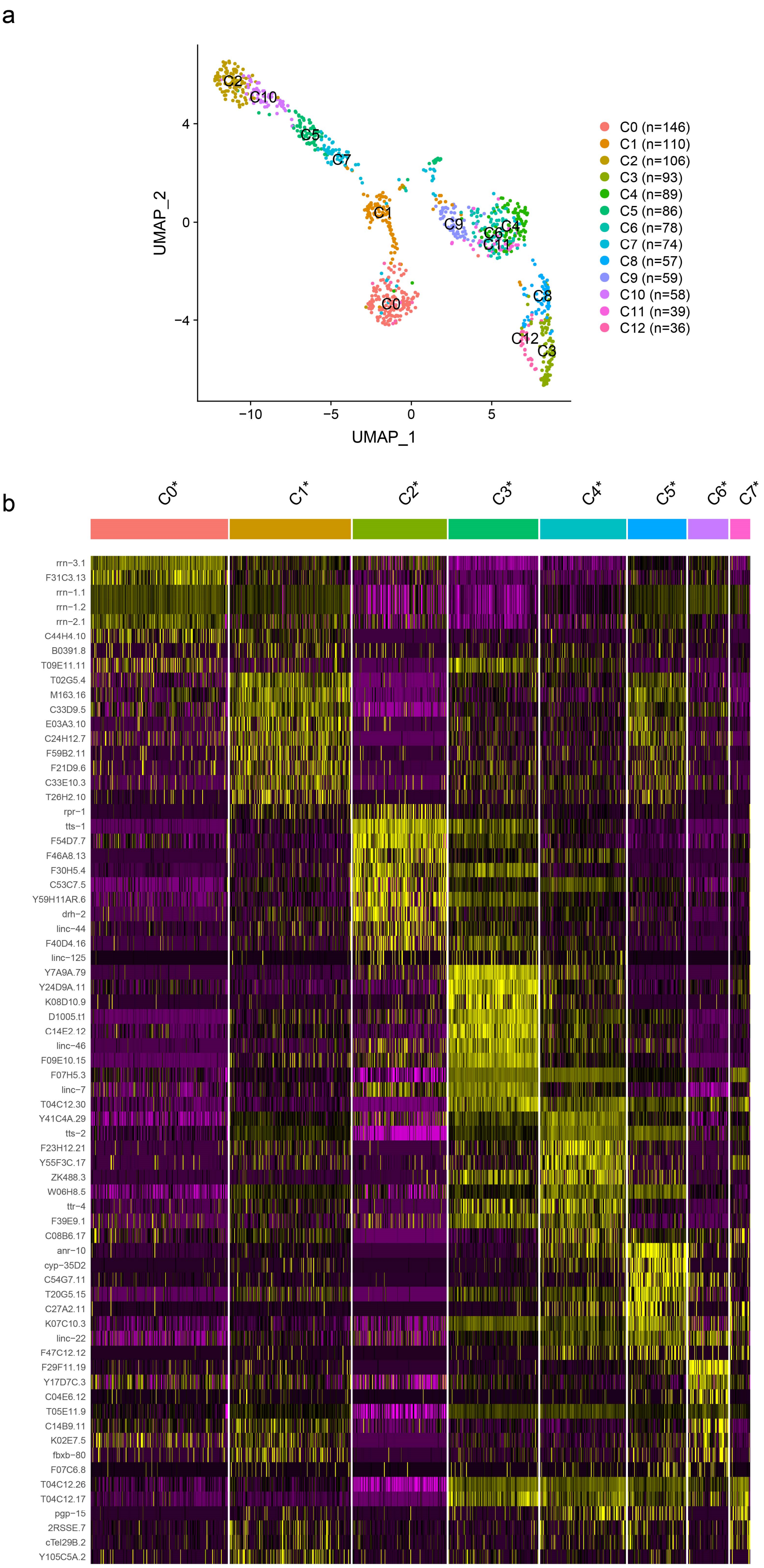
a
UMAP_2
UMAP_1
4
0
−4
−10
−5
0
5
C2
C10
C5
C7
C1
C0
C9
C6
C4
C11
C8
C12
C3
C0 (n=146)
C1 (n=110)
C2 (n=106)
C3 (n=93)
C4 (n=89)
C5 (n=86)
C6 (n=78)
C7 (n=74)
C8 (n=57)
C9 (n=59)
C10 (n=58)
C11 (n=39)
C12 (n=36)
b
C0*
C1*
C2*
C3*
C4*
C5*
C6*
C7*
rrn−3.1
F31C3.13
rrn−1.1
rrn−1.2
rrn−2.1
C44H4.10
B0391.8
T09E11.11
T02G5.4
M163.16
C33D9.5
E03A3.10
C24H12.7
F59B2.11
F21D9.6
C33E10.3
T26H2.10
rpr−1
tts−1
F54D7.7
F46A8.13
F30H5.4
C53C7.5
Y59H11AR.6
drh−2
linc−44
F40D4.16
linc−125
Y7A9A.79
Y24D9A.11
K08D10.9
D1005.t1
C14E2.12
linc−46
F09E10.15
F07H5.3
linc−7
T04C12.30
Y41C4A.29
tts−2
F23H12.21
Y55F3C.17
ZK488.3
W06H8.5
ttr−4
F39E9.1
C08B6.17
anr−10
cyp−35D2
C54G7.11
T20G5.15
C27A2.11
K07C10.3
linc−22
F47C12.12
F29F11.19
Y17D7C.3
C04E6.12
T05E11.9
C14B9.11
K02E7.5
fbxb−80
F07C6.8
T04C12.26
T04C12.17
pgp−15
2RSSE.7
cTel29B.2
Y105C5A.2

Figure S3. Clustering of the embryonic cells. (a) Cells are labelled with the clusters identified by combined protein-coding genes and ncRNAs (Fig. 2a). (b) The top 10 ncRNA markers of the eight clusters identified by ncRNAs alone.

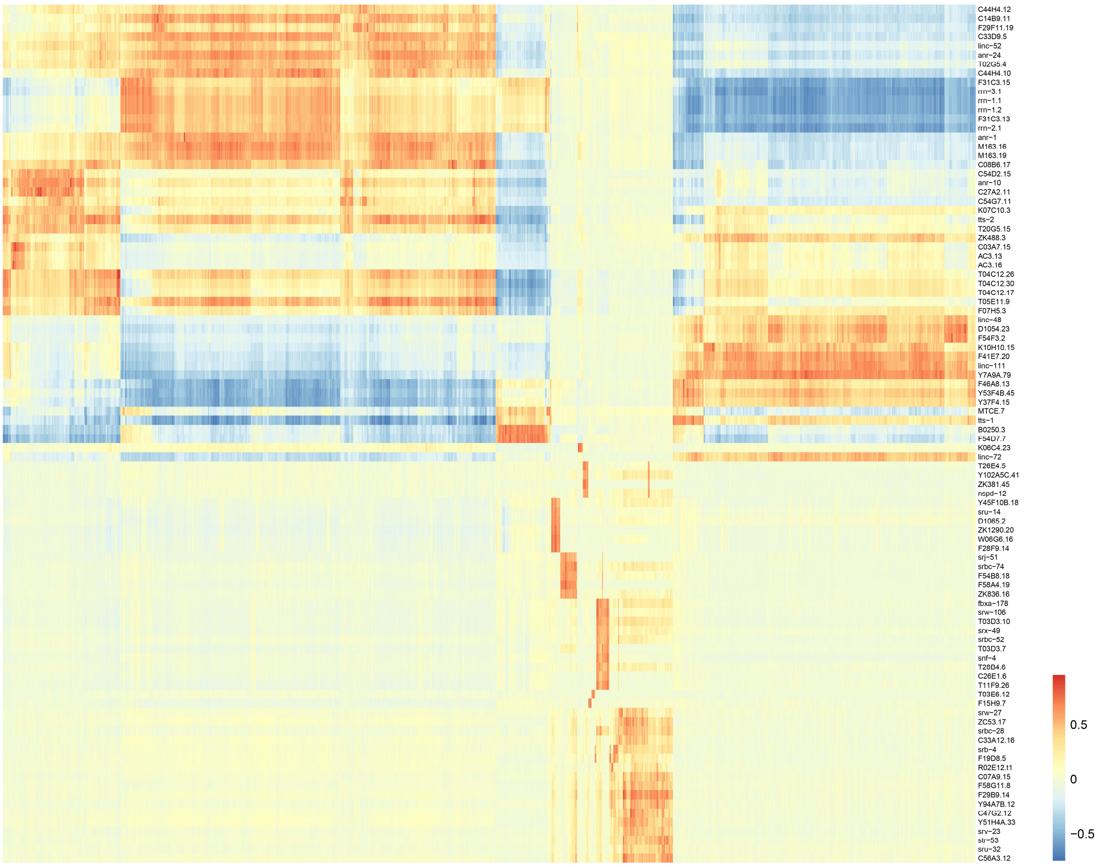

Figure S4. Heatmap of ncRNA/protein-coding gene Pearson correlations. Rows are the 94 ncRNAs, and columns are the 1208 protein-coding genes, the color represents the Pearson correlation (R). ncRNAs and protein-coding genes are hierarchical clustered.

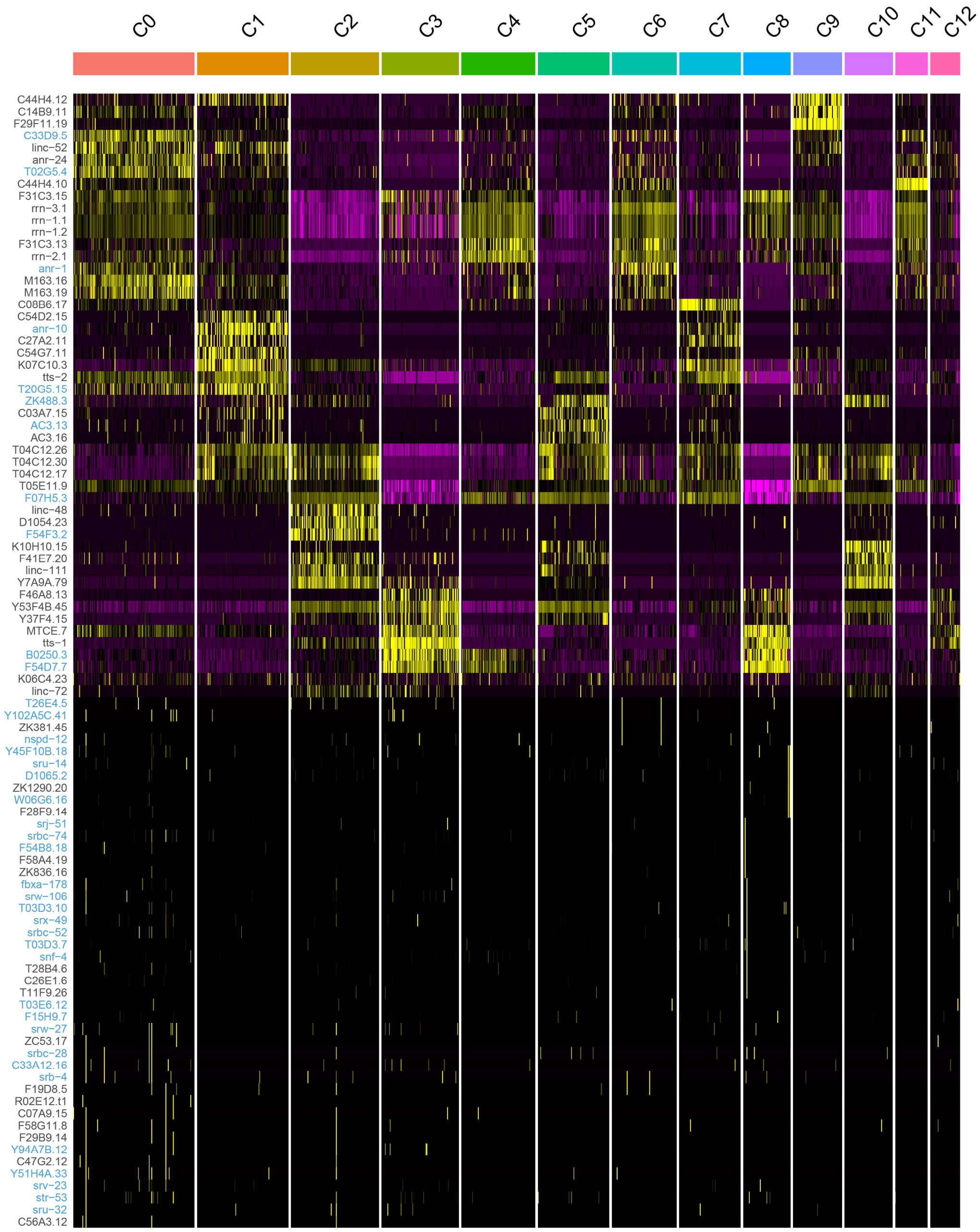

Figure S5. Heatmap of the 94 ncRNAs. Most of the ncRNAs showed cluster-specific expressions, except for most pseudogenes (terms in blue).

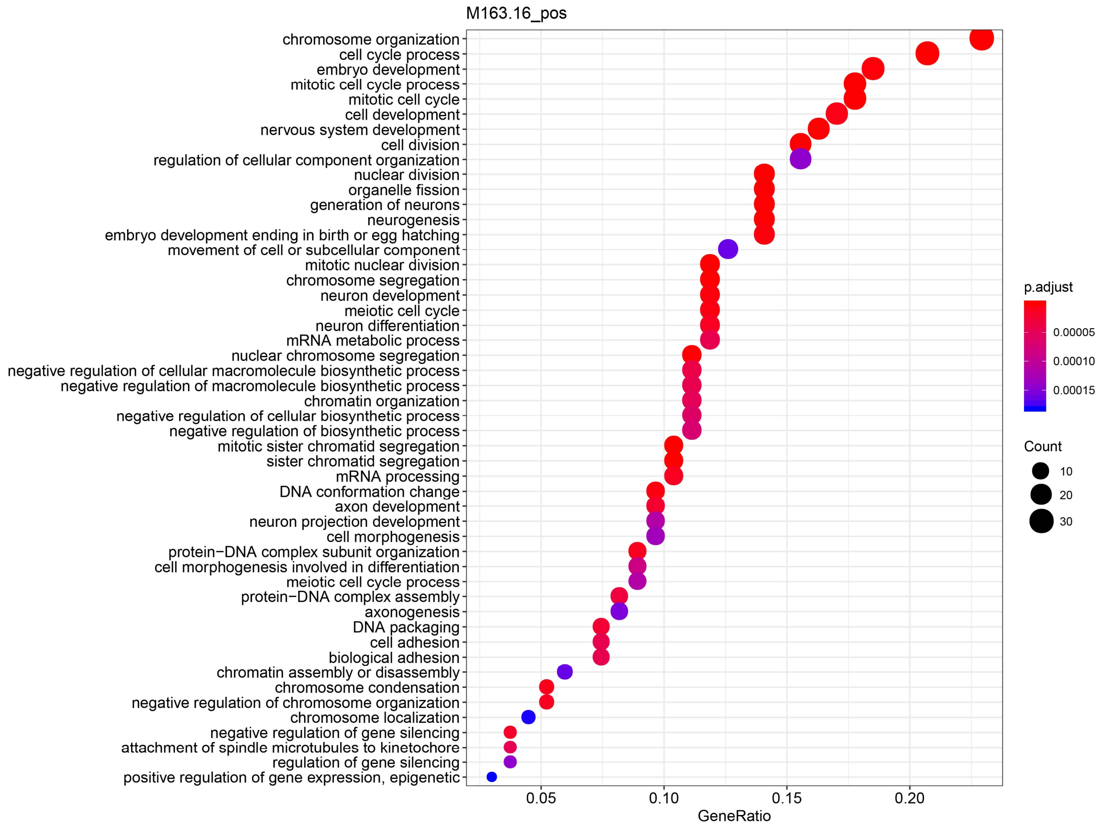

Figure S6. The top 50 enriched GO terms of the 211 protein-coding genes that are positively co-expressed with ncRNA *M163.16*.

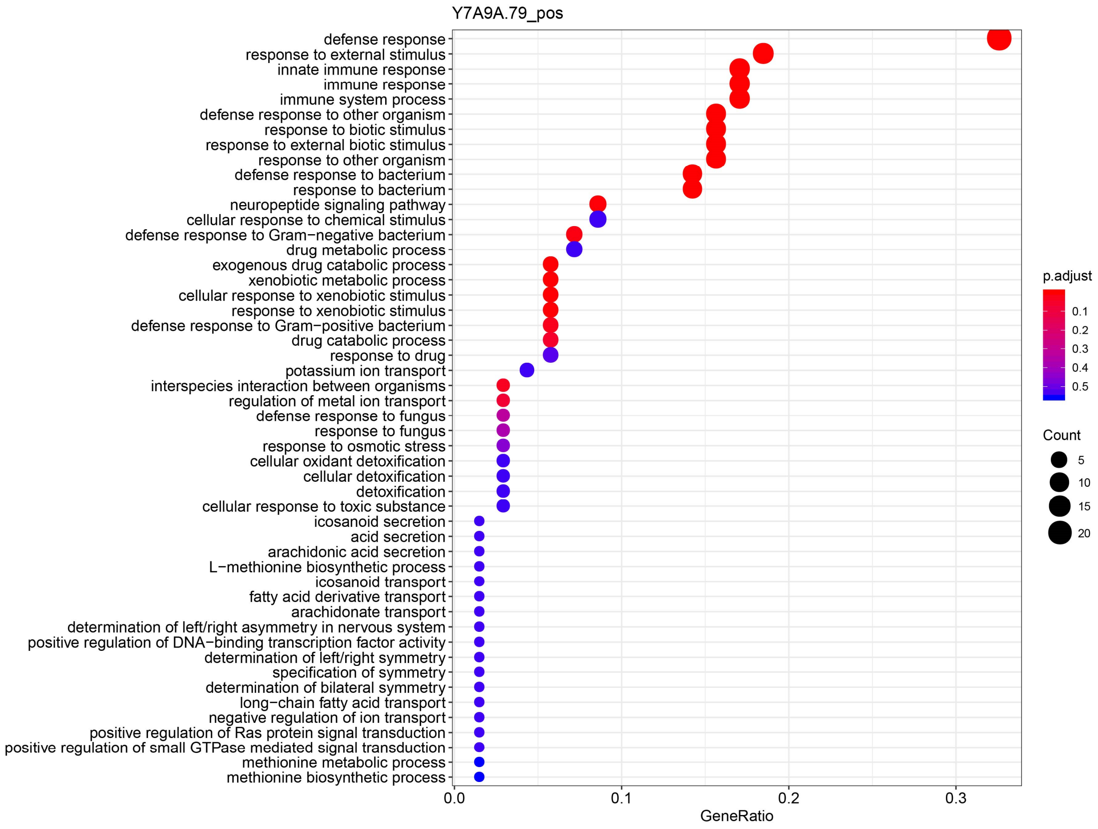

Figure S7. The top 50 enriched GO terms of the 268 protein-coding genes that are positively co-expressed with ncRNA *Y7A9A.79*.

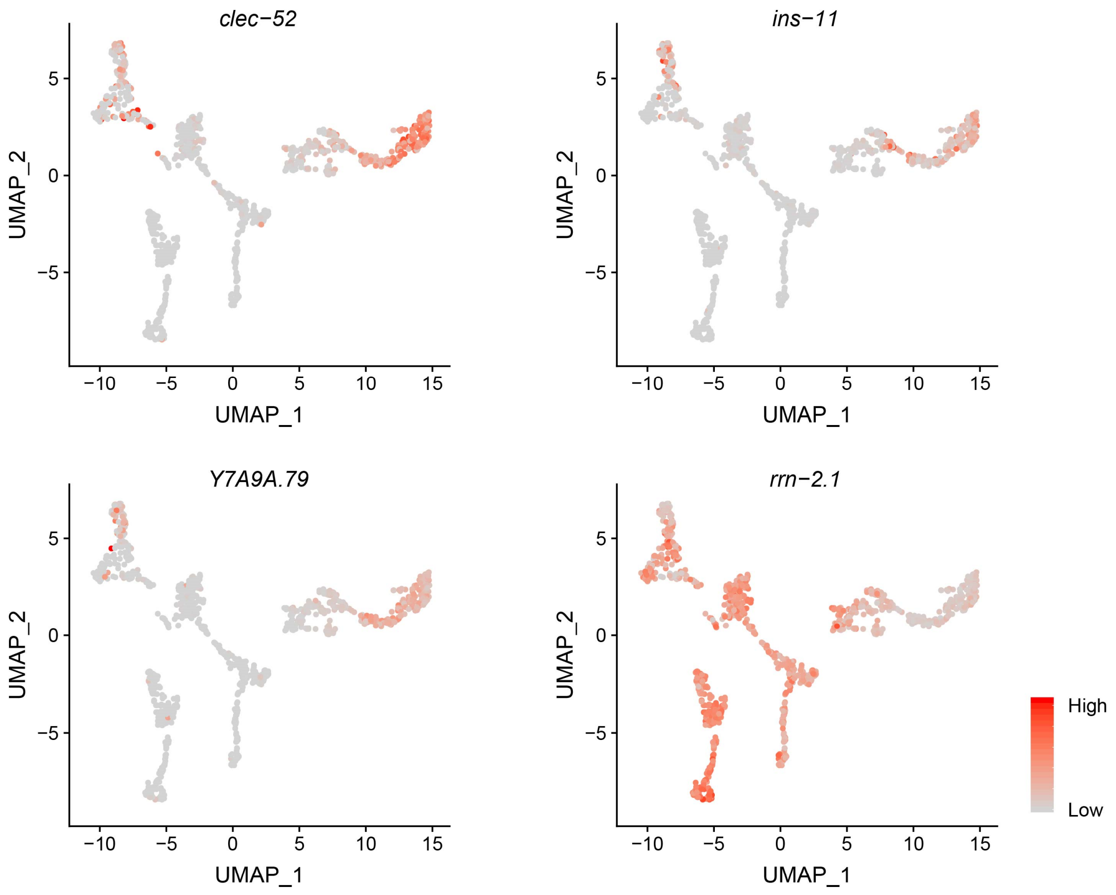

Figure S8. Feature plots showing expression levels of protein-coding genes *clec-52*, *ins-11* and ncRNAs *Y7A9A.79*, *rrn-2.1*. Protein-coding genes *clec-52*, *ins-11* are positively co-expressed with ncRNA *Y7A9A.79* and negatively co-expressed with ncRNA *rrn-2.1*.

Supplementary tables

ncRNA	ncRNA_type	Number of co-expressed coding genes with R > 0.6	Number of co-expressed coding genes with R > 0.7	Number of co-expressed coding genes with R > 0.8	Number of co-expressed coding genes with R > 0.9	Number of co-expressed coding genes with R < -0.6
anr-1	antisense	77	1	1	1	0
anr-10	antisense	71	4	1	1	0
D1054.23	unknow	67	6	1	1	0
B0250.3	pseudogene	53	12	1	1	0
C27A2.11	unknow	50	12	3	1	0
T04C12.26	unknow	49	8	4	1	3
C08B6.17	unknow	40	10	1	1	1
Y7A9A.79	unknow	268	36	1	0	1
M163.16	unknow	211	23	1	0	0
T05E11.9	unknow	155	21	1	0	4
anr-24	antisense	154	8	0	0	0
F41E7.20	unknow	127	4	0	0	0
linc-111	lincRNA	122	2	0	0	0
tts-2	unknow	116	23	0	0	2
C33D9.5	pseudogene	115	0	0	0	0
C44H4.10	unknow	96	14	1	0	0
M163.19	unknow	95	4	0	0	0
C14B9.11	unknow	87	4	1	0	0
F54D7.7	pseudogene	55	20	0	0	5
F29B9.14	unknow	42	9	1	0	0
rrn-1.1	rRNA	40	2	0	0	60
rrn-1.2	rRNA	40	2	0	0	60
F54F3.2	pseudogene	38	3	1	0	0
rrn-3.1	rRNA	37	7	0	0	113
rrn-2.1	rRNA	30	1	0	0	68
tts-1	unknow	27	3	0	0	73
F31C3.15	unknow	26	3	0	0	26
F29F11.19	unknow	26	1	0	0	0
K10H10.15	antisense	25	3	1	0	0
C54G7.11	unknow	22	0	0	0	0
C54D2.15	unknow	21	0	0	0	0
T04C12.30	unknow	17	4	2	0	7
F58A4.19	unknow	17	5	1	0	0
C56A3.12	unknow	17	2	0	0	0
C03A7.15	unknow	16	7	3	0	0
Y53F4B.45	unknow	15	3	2	0	13

srbc-74	pseudogene	12	2	0	0	0
W06G6.16	pseudogene	11	5	0	0	0
T11F9.26	unknow	11	2	0	0	0
ZC53.17	unknow	11	2	0	0	0
snf-4	pseudogene	10	1	0	0	0
C47G2.12	unknow	10	3	1	0	0
T04C12.17	unknow	10	3	0	0	0
T02G5.4	pseudogene	9	0	0	0	0
str-53	pseudogene	9	3	0	0	0
Y51H4A.33	pseudogene	9	0	0	0	0
F07H5.3	pseudogene	9	0	0	0	0
F28F9.14	unknow	8	5	0	0	0
ZK1290.20	unknow	8	3	0	0	0
srw-106	pseudogene	8	4	0	0	0
Y94A7B.12	pseudogene	8	2	0	0	0
fbxa-178	pseudogene	8	4	0	0	0
F31C3.13	unknow	7	0	0	0	0
ZK381.45	unknow	7	2	0	0	0
D1065.2	pseudogene	7	1	0	0	0
F58G11.8	unknow	7	0	0	0	0
T20G5.15	pseudogene	7	0	0	0	0
linc-52	lincRNA	6	1	0	0	0
MTCE.7	rRNA	6	0	0	0	0
srb-4	pseudogene	6	2	0	0	0
sru-14	pseudogene	6	1	0	0	0
T03D3.7	pseudogene	6	1	0	0	0
T03D3.10	pseudogene	6	1	0	0	0
F19D8.5	unknow	6	1	0	0	0
K06C4.23	unknow	6	4	0	0	0
AC3.16	unknow	6	2	2	0	0
srbc-28	pseudogene	5	0	0	0	0
C26E1.6	unknow	5	0	0	0	0
F54B8.18	pseudogene	5	1	0	0	0
ZK836.16	unknow	5	0	0	0	0
sru-32	pseudogene	5	0	0	0	0
srv-23	pseudogene	5	0	0	0	0
Y102A5C.41	pseudogene	5	0	0	0	0
linc-48	lincRNA	5	0	0	0	0
linc-72	lincRNA	5	0	0	0	0
AC3.13	pseudogene	5	0	0	0	0
K07C10.3	unknow	5	0	0	0	0
C44H4.12	unknow	4	1	0	0	0
Y45F10B.18	pseudogene	4	0	0	0	0

C07A9.15	unknow	4	0	0	0	0
srw-27	pseudogene	4	0	0	0	0
srx-49	pseudogene	4	0	0	0	0
T03E6.12	pseudogene	4	1	0	0	0
F15H9.7	pseudogene	4	1	0	0	0
srbc-52	pseudogene	4	0	0	0	0
R02E12.t1	tRNA	4	0	0	0	0
C33A12.16	pseudogene	4	0	0	0	0
T28B4.6	unknow	4	1	0	0	0
T26E4.5	pseudogene	4	2	0	0	0
nspd-12	pseudogene	4	0	0	0	0
srj-51	pseudogene	4	0	0	0	0
ZK488.3	pseudogene	4	0	0	0	0
F46A8.13	unknow	3	1	1	0	11
Y37F4.15	unknow	3	1	0	0	8

Table S1. Numbers of co-expressed protein-coding genes of the 94 ncRNAs at different cut offs of Pearson correlation (R). ncRNAs in blue are co-expressed with more protein-coding genes negatively than positively. Seven ncRNAs were co-expressed with protein-coding genes with $R > 0.9$.

ncRNA1	ncRNA2	correation	p-value	ncRNA1	ncRNA2	correation	p-value
rrn-1.1	rrn-1.2	0.99993203	0	Y7A9A.79	F31C3.15	-0.6112374	0
rrn-1.1	rrn-2.1	0.85566248	0	F31C3.13	F31C3.15	0.64613356	0
rrn-1.2	rrn-2.1	0.85547601	0	sru-32	C56A3.12	0.69669586	0
rrn-1.1	rrn-3.1	0.91829737	0	str-53	C56A3.12	0.664568	0
rrn-1.2	rrn-3.1	0.91817701	0	srbc-28	C56A3.12	0.62874053	0
rrn-2.1	rrn-3.1	0.85960099	0	F29B9.14	C56A3.12	0.71796535	0
snf-4	srw-106	0.69924867	0	snf-4	T11F9.26	0.74324066	0
srw-106	srx-49	0.63096136	0	srw-106	T11F9.26	0.72822075	0
snf-4	srbc-52	0.61208274	0	srx-49	T11F9.26	0.60912872	0
tts-2	T05E11.9	0.63157825	0	srbc-52	T11F9.26	0.60920923	0
F07H5.3	T05E11.9	0.61562624	0	fbxa-178	T11F9.26	0.66328126	0
snf-4	fbxa-178	0.64723151	0	T03D3.7	T11F9.26	0.68535375	0
srw-106	fbxa-178	0.7734674	0	T03D3.10	T11F9.26	0.63290427	0
srx-49	fbxa-178	0.65934002	0	C26E1.6	T11F9.26	0.67908167	0
B0250.3	F54D7.7	0.70008587	0	sru-14	ZK1290.20	0.70699741	0
sru-14	D1065.2	0.65519831	0	D1065.2	ZK1290.20	0.70822522	0
snf-4	T03D3.7	0.65383248	0	Y45F10B.18	ZK1290.20	0.61989309	0
srw-106	T03D3.7	0.64607128	0	W06G6.16	ZK1290.20	0.82900613	0
snf-4	T03D3.10	0.62124729	0	Y94A7B.12	C07A9.15	0.7407668	0
srw-106	T03D3.10	0.69509307	0	F29B9.14	C07A9.15	0.75297142	0
srx-49	T03D3.10	0.61661403	0	AC3.13	AC3.16	0.63210857	0
srbc-52	T03D3.10	0.64904506	0	C03A7.15	AC3.16	0.6361805	0
fbxa-178	T03D3.10	0.73056286	0	tts-2	T04C12.26	0.68950594	0
C33A12.16	srbc-28	0.61933927	0	F07H5.3	T04C12.26	0.63442026	0
fbxa-178	srbc-28	0.60235829	0	T05E11.9	T04C12.26	0.70193437	0
T03D3.10	srbc-28	0.63805681	0	T04C12.17	T04C12.26	0.76293805	0
srbc-74	F54B8.18	0.67695387	0	srbc-74	ZK836.16	0.66974178	0
sru-14	W06G6.16	0.74059563	0	F54B8.18	ZK836.16	0.68591825	0
D1065.2	W06G6.16	0.79865896	0	F58A4.19	ZK836.16	0.66613015	0
Y45F10B.18	W06G6.16	0.65109622	0	tts-2	C08B6.17	0.64559694	0
str-53	Y94A7B.12	0.6033907	0	C27A2.11	C54D2.15	0.64283852	0
rrn-1.1	Y7A9A.79	-0.6593736	0	sru-14	F28F9.14	0.73879253	0
rrn-1.2	Y7A9A.79	-0.6589093	0	D1065.2	F28F9.14	0.73896214	0
rrn-2.1	Y7A9A.79	-0.6287487	0	Y45F10B.18	F28F9.14	0.64735355	0
rrn-3.1	Y7A9A.79	-0.6383281	0	W06G6.16	F28F9.14	0.86050264	0
rrn-1.1	F31C3.13	0.72310906	0	ZK1290.20	F28F9.14	0.82224064	0
rrn-1.2	F31C3.13	0.72331664	0	srw-27	ZC53.17	0.71911004	0
rrn-2.1	F31C3.13	0.75560002	0	C33A12.16	ZC53.17	0.63090273	0
rrn-3.1	F31C3.13	0.76838723	0	srbc-28	ZC53.17	0.67461908	0
Y51H4A.33	C47G2.12	0.61521184	0	Y51H4A.33	ZC53.17	0.60882716	0
Y94A7B.12	C47G2.12	0.65603179	0	tts-2	T04C12.30	0.6065825	0
snf-4	C26E1.6	0.65012133	0	T05E11.9	T04C12.30	0.6018009	0
srw-106	C26E1.6	0.67335574	0	F54D7.7	T04C12.30	-0.6472885	0
str-53	F29B9.14	0.7429714	0	T04C12.17	T04C12.30	0.88755986	0
Y51H4A.33	F29B9.14	0.60568333	0	T04C12.26	T04C12.30	0.82546294	0
Y94A7B.12	F29B9.14	0.83168833	0	Y7A9A.79	F41E7.20	0.63086235	0
C47G2.12	F29B9.14	0.65140901	0	Y7A9A.79	linc-111	0.67588921	0
Y94A7B.12	F58G11.8	0.62194991	0	F41E7.20	linc-111	0.60930915	0
F29B9.14	F58G11.8	0.72970952	0	T20G5.15	anr-10	0.6249538	0
srj-51	F58A4.19	0.80034732	0	C27A2.11	anr-10	0.60815432	0
srbc-74	F58A4.19	0.70812233	0	F54F3.2	linc-48	0.60591262	0
F54B8.18	F58A4.19	0.71363293	0	T02G5.4	anr-24	0.66728379	0
T26E4.5	ZK381.45	0.74990421	0	M163.16	anr-24	0.62891786	0
M163.16	M163.19	0.81224315	0	F54F3.2	D1054.23	0.69175615	0
rrn-1.1	F31C3.15	0.89781227	0	tts-1	F46A8.13	0.60215364	0
rrn-1.2	F31C3.15	0.89790626	0	Y53F4B.45	F46A8.13	0.67405603	0
rrn-2.1	F31C3.15	0.79426844	0	Y53F4B.45	Y37F4.15	0.70156101	0
rrn-3.1	F31C3.15	0.89963345	0	F46A8.13	Y37F4.15	0.64189397	0

Table S2. Pearson correlations between each two of the 94 ncRNAs. Only those whose absolute

values of the Pearson correlations (R) are greater than 0.6 are listed. Terms in red are co-expressed ncRNA pairs mentioned.
